# Real-life experience of accepting assistive device services for Tibetans with dysfunction: A qualitative study

**DOI:** 10.1016/j.ijnss.2022.12.005

**Published:** 2022-12-21

**Authors:** Jun Luo, Zhujizhaba Gama, Deji Gesang, Qing Liu, Ying Zhu, Lining Yang, Dingqun Bai, Mingzhao Xiao

**Affiliations:** a Department of Urology, The First Affiliated Hospital of Chongqing Medical University, Chongqing, China; b Department of Rehabilitation Medicine, Tibet Autonomous Region People’s Hospital, Tibet, China; c Department of Rehabilitation Medicine, The First Affiliated Hospital of Chongqing Medical University, Chongqing, China

**Keywords:** Experience, Ethnic and Racial Minorities, Qualitative research, Self-help devices

## Abstract

**Objective:**

This study aimed to understand the real-life experiences of Tibetans in China with dysfunction in the process of accepting assistive device services and to provide a reference for service quality improvement and policy formulation.

**Methods:**

Semi-structured personal interviews were used to collect data. Ten Tibetans with dysfunction representing three categories of different economic level areas in Lhasa, Tibet were selected to participate in the study by purposive sampling method from September to December 2021. The data were analyzed using Colaizzi’s seven-step method.

**Results:**

The results present three themes and seven sub-themes: identification of tangible benefits from assistive devices (enhancing self-care ability for persons with dysfunction, assisting family members with caregiving and promoting harmonious family relationships), problems and burdens (difficulty in accessing professional services and cumbersome processes, not knowing how to use it correctly, psychological burden: fear of falling and stigmatization), and needs and expectations (providing social support to reduce the cost of use, enhancing the accessibility of barrier-free facilities at the grassroots level and improving the environment for the use of assistive devices).

**Conclusion:**

A proper understanding of the problems and challenges faced by Tibetans with dysfunction in the process of accepting assistive device services, focusing on the real-life experiences of people with functional impairment, and proposing targeted suggestions for improving and optimizing the user experience can provide reference and basis for future intervention studies and related policy formulation.

## What is known?

• Assistive devices can help persons with dysfunction improve their self-care ability and quality of life.
• Improving the service quality of assistive devices is conducive to actively responding to the aging population and taking proactive health actions.
• Tibetans have limited access to assistive device services, and the status of these services could be better.

## What is new?

• The study revealed the current situation of assistive device services in Tibet Autonomous Region, China and the real-life experiences, feelings, and needs of Tibetan people with dysfunction in accepting assistive device services.
• Religious beliefs have little effect on the acceptance of assistive device services by residents in Tibet, where the entire population identifies as religious.

## 1 Introduction

China has more than 85 million people with disability [1] and more than 260 million elder people aged 60 years and over [2]; the increasingly serious aging problem and the huge number of people with disability have made China the country with the largest demand for assistive devices and the largest market potential in the world [3]. The demand for assistive devices from the vast number of elder and disabled groups is extremely urgent [4]. Assistive devices (ADs) are products (including instruments, apparatus, equipment, and software) used by people with dysfunction to prevent injury, compensate for somatic functions, monitor daily activities, reduce or offset limitations, and improve social life participation [5]. ADs enable the users to maintain independence for a longer period, including seeking medical treatment, assuming family responsibilities, and participating in social events [6]. Walking sticks, wheelchairs, hyperopic spectacles, reading glasses, and dentures are examples of common ADs [5].

Tibet Autonomous Region is located in the Qinghai-Tibet Plateau with a high altitude, low oxygen content, and harsh climate, and residents like to eat beef, mutton, butter tea, and other high-purine and high-fat foods, resulting in a high incidence of stroke in Tibet [7]. However, owing to poor local medical conditions [8], patients are often not treated on time, resulting in dysfunction or even disability [9]. In addition, most residents in local areas continue to follow traditional production methods and long-term heavy workloads, which also cause greater damage to the weight-bearing joints of residents, and the chance of dysfunction is higher than that in the plains. The risk factors of dysfunction, combined with the large population aged or with disability (342,000) [10], show a huge demand for ADs services. However, because the per capita disposable income of residents [11] is much lower than the national average [12], coupled with barriers of traffic and language, the development of ADs services and scientific research are difficult, and relevant data are scarce. In previous studies, we learned that the development of ADs in China is still facing problems of great differences in regional services, non-standard adaptation services, and unsound service systems [13]. Existing studies focus on Eastern China [14], Central China [15], and other provinces and cities in Western China [16], while the current situation of ADs services in Tibet need to be paid attention to. Therefore, this study conducted a qualitative study on Tibetan ADs users to understand their real experiences and needs in the process of accepting ADs services to provide reference suggestions for improving the quality of ADs services in the area.

## 2 Methods

### 2.1 Study design

A descriptive qualitative study was conducted to explore the real-life experience of accepting ADs services for Tibetans with dysfunction through individual face-to-face semi-structured interviews [17]. This study followed the Consolidated Criteria for Reporting Qualitative Studies (COREQ).

### 2.2 Setting and participants

A purposive sampling method was employed to identify Tibetans with dysfunction. Participants were recruited from three different districts of Lhasa City in the Tibet Autonomous Region: Cemenlin Community, Jiaerxi, and Cijuelin Village, representing urban, rural, agricultural, and pastoral areas of different economic levels, from September 2021 to December 2021. Inclusion criteria were as follows: 1) conform to types of functional impairment listed in the national standard “Classification and grading criteria of disability” (GB/T26341-2010) [18]; 2) identified as Tibetan; 3) has a history of using ADs, and 4) understand the purpose of the interview. Exclusion criteria: 1) combined with other serious physical diseases and inability to participate in the interview; 2) with hearing, language, or cognitive impairment and the inability to cooperate with the interviewer.

### 2.3 Recruitment and enrollment

To maximize the diversity and representativeness of the sample, participants were selected according to their residence, economic status, degree and duration of dysfunction, and type of ADs use. Two rounds of interviews were conducted with the participants. From September 21 to 27, 2021, with the help of community managers, telephone calls were made in advance with participants to determine the time and place of the interviews and to provide an initial introduction to the study. Seven participants were interviewed during the first round. Following the methodology proposed by Francis et al. [19], we planned a second round of interviews to be conducted when the research team confirmed that saturation had been reached and no new themes were emerging from the data. With this approach, we conducted a second interview on December 1, 2021, and contacted the interviewees by telephone to communicate the time, place, and research background. When two round of interviews were completed, no new topics emerged, and thus we chose to end the interviews.

### 2.4 Ethical considerations

This study was approved by the ethics committee of the First Affiliated Hospital of Chongqing Medical University (approval number 2020–622). All participants were informed of the aims of the study, the procedure, and their right to refuse participation and withdraw at any period in the research process. All participant information was anonymized to protect their privacy. All participants signed an informed consent form to voluntarily participate in this study.

### 2.5 Data collection

Semi-structured interviews were used to collect data. The researcher reviewed information on the regional disease spectrum, religious beliefs, and local policy documents in the Tibet Autonomous Region before the formal interviews to fully understand Tibetan culture. Based on the purpose of the study, an interview outline was initially informed by a literature review, and two cases were selected for a pilot interview. Five clinical experts modified the final interview outline. These experts had a bachelor’s degree or above, working in a tertiary hospital in Tibet, had been engaged in rehabilitation therapy technology for more than 10 years, and had experience providing ADs services. All interviews were conducted in either Tibetan or Mandarin, depending on the language usage habits of the participants. The main contents of the interview outline included: 1) What changes did the use of ADs bring to your life? 2) What do you think about your need to use ADs? 3) Can you discuss the process of configuring ADs? 4) What feelings did you have during the process of using ADs? What difficulties did you encounter? 5) Did you have any other suggestions, expectations, or additions to this conversation?

Interviewees were selected in strict accordance with the inclusion and exclusion criteria. The purpose and content of these were explained, and a time was agreed upon for the interviews with the consent of the participants. The interviews were conducted in a separate meeting room of the community/village council to avoid interference. The researcher remained neutral throughout the interview, using techniques such as listening, appropriate follow-up questions, rhetorical questions, clarification, repetition, and response while taking care not to influence the interviewer’s thinking and expression. Second, the researcher aimed to encourage the interviewees to fully express their true thoughts and carefully observe and record nonverbal information during the interview, including interviewees’ expressions and body language. The duration of each interview was limited to 30–40 min. All interviews were recorded using the cell phone recording function and were kept strictly confidential. Data collection was complete when saturation was reached.

### 2.6 Data analysis

The interviews were transcribed verbatim within 24 h of completion. Two researchers (J. Luo and Y. Zhu) listened to the recordings repeatedly until they had a good understanding of the overall content. The data were analyzed using the Colaizzi’s seven-step analysis [20]: 1) familiarization with the data through multiple readings of the transcripts; 2) identification of significant statements; 3) formulating meanings based on a careful review of significant statements; 4) clustering meanings into themes; 5) developing an inclusive description of the phenomenon based on themes; 6) condensing exhaustive descriptions down to short statements that capture the essential structure of the phenomena, and 7) seeking verification of the fundamental structure. Two individuals independently read the transcripts to identify all meaningful units associated with the experience of the ADs services. Throughout the process, we compared the relevance of the identified meaningful units until a consensus was reached. These units were condensed into codes based on their similarities, and themes, and sub-themes were identified. To maintain the objectivity of the analysis, the researchers analyzing the data (J. Luo and Y. Zhu) were asked to explicitly write down their pre-existing beliefs and opinions before data collection to ensure that any preunderstandings or assumptions could be set aside as much as possible. We then returned the transcribed text and results to participants for review, with the researcher (D. Gesang) responsible for objective translation and interpretation, where there was no understanding until an agreement was reached. Finally, the qualitative results were sent to qualitative experts who were not involved in the data analysis in the first stage for validation.

### 2.7 Rigor

Pilot interviews were conducted before the formal interviews, and the interview outline was adjusted based on the pilots’ results. To ensure an accurate understanding of the study participants’ statements, a Tibetan medical staff member with professional knowledge of rehabilitation, who was bilingual in Tibetan and Chinese and familiar with qualitative research methodology, was invited to serve as an interview interpreter and consultant during the interview. She was responsible for the Tibetan-Chinese translation during the interview process and, translated and interpreted the statements made by the study participants objectively based on Tibetan culture to ensure that the researcher’s understanding was free of bias.

## 3 Results

The results demonstrated that ten respondents were between 50 and 80 years old, half of them relied on agricultural income, and half had an average household monthly income of less than 1,000 yuan (Table 1). Three themes and five subthemes emerged from data analysis.

**Table 1:** Demographic data of the participants.

ID	Gender	Age (years)	Marital status	Educational background	Employment status	Household monthly income/yuan	Place of residence	Degree of dysfunction	Care situation	Type of use and time
P1	Female	73	Widowed	High school	Retirement	＞5,000	Urban	Semi-disabled	Living alone	Visual aid (10 years)
P2	Female	79	Widowed	Middle school	Farming/grazing	2,001–5,000	Urban	Semi-disabled	Living alone	Visual aid (15 years); Medical aid (3 years)
P3	Female	60	Widowed	University	Retirement	2,001–5,000	Rural	Semi-disabled	Living alone	Visual aid (5 years)
P4	Female	60	Married	Middle school	Retirement	＜1,000	Rural	Incapable	Child care	Walking stick (3 years); Wheelchair（1 year）
P5	Male	78	Married	Primary school and below	Farming/grazing	＜1,000	Rural	Disability (Level 3)	Child care	Walking stick (5 years); Wheeled walking frame (1 day)
P6	Female	63	Married	High school	Farming/grazing	1,000–2,000	Urban	Incapable	Spousal care	Wheelchair (10 months)
P7	Female	74	Widowed	Primary school and below	Retirement	1,000–2,000	Urban	Disability (Level 4)	Living alone	Walking stick (2 years); Wheeled walking frame (2 days)
P8	Male	50	Married	Middle school	Unemployed	＜1,000	Rural	Disability (Level 2)	Spousal care	Blind cane (8 years); Hand rehabilitation trainer (2 years)
P9	Female	80	Widowed	Primary school and below	Farming/grazing	＜1,000	Rural	Incapable	Child care	Walking stick (1 year); Denture (10 years)
P10	Female	54	Married	High school	Farming/grazing	＜1,000	Rural	Disability (Level 1)	Cared by relatives/friends	Denture (3 years); Visual aid (6 months)

### 3.1 Theme 1: recognition of the tangible benefit of ADs

ADs not only assist and support people with dysfunction to complete their daily activities and improve their ability to take care of themselves but also reduce the burden on caregivers and promote harmonious family relationships.

#### 3.1.1 Subtheme 1: enhance self-care ability for persons with dysfunction

Tibetans with dysfunction described how the use of ADs has made their lives easier, and they are very satisfied with this.

*“My eyes are blurry and I can’t see clearly when I watch TV without reading glasses. But with the glasses, I can see clearly.”* (P1)

*“I can take care of myself with the walking stick. Second, I can protect myself with it. If I do not use it to help me walk, it may be a little dangerous … Using the walking stick to a certain extent is to protect myself from harm …”* (P5)

*“My teeth are not good … wearing a denture, the heart is much more secure, and eating is more convenient.”* (P10)

#### 3.1.2 Subtheme 2: assist family members with caregiving and promote harmonious family relationships

Participants reported that ADs relieved the burden of care for their families that there were fewer arguments between families, and their psychological burden was reduced.

*“After I use the walking stick, I don’t need to have my family around me 24h a day. When they go out, I can use it to complete some simple movements … my children can feel more assured, and I can also take some of the burdens of my children.*” (P5)

*“Generally, when I am alone and no one helps me, I put the walking stick beside my bed, which can lighten the burden of family care and the children can concentrate on their work … I found that there were fewer quarrels in the family, the family atmosphere was more harmonious, and the family relationship was better than before.”* (P9)

### 3.2 Theme 2: problems and burdens

ADs services face problems of accessibility and professionalism in less economically developed areas and among vulnerable populations, which are also unmet needs of dysfunctional groups. In addition to providing material resources and the environment, we should also pay attention to the psychological resilience of the vulnerable population.

#### 3.2.1 Subtheme 1: difficulty in accessing professional services and cumbersome processes

Respondents’ communities/village committees lacked professional places and professional personnel to help them adapt to ADs, and the disability commissioners were non-professional part-time community workers. However, the long distance and cumbersome process of accessing professional services are barriers that affect the service experience of older people with dysfunction.

*“I always go to the store to buy it (hyperopic spectacles), and going to the hospital or professional institutions process is too troublesome. We are older … Walking is not convenient for us. We prefer to go to the store to buy and leave immediately.”* (P2)

*“If we wanted a professional to fit us individually, we would need to go to the urban disability organization, a long way from home, which was a hassle. The village council disability commissioner would come and collect our needs and then report them, without assessing or teaching us how to use them.”* (P8)

*“If I want to go to a professional institution, I need to walk to a place far away from my home to take the bus … I have to transfer once to a city bus in between, which takes nearly two hours to get there.”* (P9)

#### 3.2.2 Subtheme 2: not knowing how to use it correctly

The majority of participants choose to go to a store where the salesman is unable to provide proper instruction and training on its use and does not even mention such information, which is potentially harmful to the safety of the users. Combined with fading memory, verbal or written instructions can become blurred over time, with deviations in the information or even complete forgetfulness.

*“I buy (the walker) randomly in the street store. I am old and have a very poor memory … I cannot always remember the techniques used to exert force when using it.”* (P7)

*“When I went to the store, I said I needed a cane, and the salesman took it directly to me and didn’t give me some guidance after I bought it.”* (P9)

#### 3.2.3 Subtheme 3: psychological burden: fear of falling and stigmatization

Most participants reported that they had a history of falls and had safety concerns when using ADs. On the contrary, most of the participants experienced negative emotions of “stigmatization” of ADs as a “marker” of externalization of “disability”.

*“I’m still a little embarrassed to wear it (reading glasses) during the day, I’m too embarrassed to wear it out, so I only wear it when I am alone at home”* (P1)

*“This anti-slip pad at the bottom of the crutch is made by myself. The original one bought in the store is very slippery, and I am very afraid of falling.”* (P9)

### 3.3 Theme 3: needs and expectations

Participants were not satisfied with the current social support network, and they hoped to acquire professional support. Compared with other medical services, they would like to receive a larger percentage of government funding for ADs services and a well-developed barrier-free environment at the grassroots level for comprehensive support.

#### 3.3.1 Subtheme 1: provide social support to reduce the cost of use

Half of the interviewees have farming/grazing as their main source of income, and their incomes are unstable. They rely on their families to obtain ADs and hope that the government will provide policy and financial support. On the other hand, local medical conditions are poor, and some Tibetans go to temples for medical treatment. Misdiagnosis or inappropriate diagnosis and treatment occasionally occurs, resulting in functional impairment without improvement or permanent disability. The participants hoped that scientific adaptation could improve their physical function, self-care ability, and quality of life and avoid secondary injuries caused by non-professional adaptation.

*“It’s better to give me some professional guidance … I would like to have a professional in the community to provide services for us so that we can always consult him if we encounter any problems in the process of using it (reading visual aid).”* (P3)

*“People with disabilities generally have policy support, and older people are getting older and naturally need walkers, crutches, etc., but there are no subsidies.”* (P4)

*“My family’s financial situation is not very good, and my family belongs to a low-income household. Now I am old, I can’t walk easily and I can’t see very well. On the one hand, I hope a professional will provide me with (ADs) services, and on the other hand, I want policy and financial support to be given.”* (P6)

#### 3.3.2 Subtheme 2: enhance the accessibility of barrier-free facilities at the grassroots level and improve the environment for the use of ADs

In addition to configuring ADs according to the user’s physical impairment, the assessment of the environment of use is also important [21]. Second, building an environment for the use of ADs and barrier-free facilities at the grassroots level can improve the social participation and well-being of socially disadvantaged groups [22].

*“I have a mobility impairment and so does my wife. Every time we go up a slope is difficult … We need to climb stairs, and the pavement is so potholed that walkers with wheels underneath do not work at all; we both want a flat surface and have handrails to hold us up the slope.”* (P5)

*“Can the community have an elevator? We would like to go up to the second floor even if we are in wheelchairs.”* (P6)

*“I previously bought a walker with wheels at random, the shopkeeper did not ask me whether the living environment is suitable or not … I bought it back only to find that the village road is not at all suitable for its use.”* (P7)

## 4 Discussion

### 4.1 Multi-dimensional service experience of ADs

ADs improve users’ ability to take care of themselves [23,24]. Dysfunction was the main factor influencing the use of ADs amongst all participants, as mentioned in several previous studies [[25], [26], [27]]. All participants reported an increase in their self-care ability after use. On the other hand, ADs also became an important tool to satisfy the sense of self-worth [28]. When experiencing sudden functional impairment or disability, the sense of self-worth can decline rapidly [29,30]. ADs can enable people with dysfunction to return to their family and social roles and compensate for their lack of self-worth [31,32]. However, many problems affect the experiences of people with dysfunctions in the process of use. Due to geographical and economic constraints, there are regional differences in public health services and unequal services in rural and remote areas [33]. The difficulty in accessing professional services and the cumbersome application process has significantly increased insecurity. For a long time, the social perception of ADs as an externalized label of “disability” has made most people with dysfunction have a negative experience of “stigma” in public places [34,35]. Another group of participants reported that they had a history of falls, and the use of ADs created a sense of “fear” for them [36,37]. In addition, due to the non-professional configuration of ADs, most participants reported that they did not receive any instruction and training and often felt frustrated when using them, which made them consider abandoning them.

### 4.2 Religious belief is not a barrier to the use of ADs

Tibet is a region where almost everyone has religious beliefs, and Tibetans have devout beliefs that influence their health beliefs and behaviors [38,39]. In the process of interviewing, we found that most participants would choose to go to a temple near their community and ask the lamas for help before using ADs. They hope to be blessed by Buddha, but the results are often unsatisfactory. Interviewees found that their functional impairments were becoming more severe, even leading to complete loss of function and permanent disability. This prompts Tibetans to go to professional institutions for treatment and to be equipped with appropriate ADs to compensate for or replace the loss of function. This is where indigenous religious and cultural preferences become an inducing factor for accepting ADs services, which is one of the influencing factors for their positive attitudes toward the majority of ADs. Additionally, they may express strong resistance to certain types of ADs that appear in specific scenarios, such as nursing beds used in medical institution settings. Utilizing this type of ADs is seen as an ominous sign and they refuse to use them even though they need them so urgently, which may be related to the vulnerability of plateau areas to poor psychological conditions and the existence of certain social prejudice and discrimination [40]. In the future, relevant research can invite experts in the fields of spirit, psychology, and religion and some scholars with religious beliefs to join in, and make use of the local religious culture to carry out characteristic ADs services, so that vulnerable groups in plateau areas have a wider acceptance of ADs and better health outcomes.

### 4.3 Countermeasures

#### 4.3.1 Enhance the professionalism and accessibility of grassroots services

Nonprofessional services can cause secondary damage to users [41,42]. In this study, the participants’ community/village committees could not provide accessible professional services, and most of the participants chose to make their own or purchase from stores that were closer and more convenient but professionally unqualified, which posed a threat to users’ safety. However, advice and guidance from professionals would promote their willingness to use and improve compliance [43,44]. Given the above, official organizations can carry out professional training for grassroots disability commissioners, strictly screen the qualifications of grassroots professionals, assign a commissioner to be responsible for adapting ADs in specific areas, and consider professionalism while taking into account the accessibility of services.

#### 4.3.2 Strengthen the scientific popularization of ADs and reduce the psychological burden

People who use ADs are more likely to have a fear of falling than non-users [45,46], which is consistent with the results of this study. This study found that some participants had a history of falls, were uncertain about the function and safety of ADs when they first used them, and were afraid of falling again. In addition, most of the participants had low self-esteem during the use of ADs and felt like someone was talking about them behind their backs in public, creating a heavier psychological burden. This finding was also found by Williams et al. [47]. In the process of ADs service provision, in addition to paying attention to the physical impairment of the user, their psychological condition should not be ignored. Therefore, it is necessary to carry out health education programs on ADs for individuals and families for society as a whole. Considering the Tibetan religious and cultural background and language usage habits, we should strengthen the scientific popularization and professional guidance of ADs among the applicable population and gradually improve the correct understanding of ADs among the public to reduce the psychological pressure caused by society on the users of ADs.

#### 4.3.3 Integrate external resources and strengthen social support

Dysfunctional groups are often socially disadvantaged and have limited ability to access resources on their own [48]. In this study, most participants with dysfunction wanted multifaceted support from family, professionals, the government, and society. Financial, emotional, and active support from family members would be beneficial for improving the coping abilities of people with dysfunction. Participants indicated that the lack of a supportive environment in their community was one of the leading factors for high abandonment. Improving accessibility facilities and infrastructure in the grassroots community is a prerequisite for dysfunctional groups to participate in social life. In addition, the coverage and extent of government welfare policies and funds are the most important concerns for people with dysfunction and their families. Owing to insufficient economic development and the weak resilience of individuals and families to resist risks, it is mostly supported and subsidized by the government in Tibet. Policy and funding support for ADs has not yet been perfected. There is only one policy of distribution for some people with disabilities, and professionalism cannot be guaranteed. However, the welfare policy of ADs for the elderly has not yet been introduced. As the national aging process accelerates, the role played by ADs in actively coping with the aging problem is becoming increasingly prominent, and the gradual coverage of the welfare policy of ADs for the elderly with dysfunction is a key direction to be urgently explored in our future research.

### 4.4 Limitations

The study was conducted in the unique cultural context of Tibet, and the interviewees were all ethnic minorities in Lhasa, which may limit the generalizability of the results. In the future, we hope to explore ADs service utilization status in various ethnic groups to meet their demands.

## 5 Conclusions

This study explored the real-life experiences of Tibetans with dysfunction in the process of accepting ADs services in a cross-cultural context through semi-structured interviews and summarized the problems and burdens faced by Tibetans with dysfunction in the course of services and needs/expectations. Future interventions should focus on enhancing the professionalism and accessibility of ADs services at the grassroots level, strengthening the scientific popularization of ADs, focusing on the psychology of people with dysfunction, and integrating external resources to provide comprehensive social support for people with dysfunction.

## CRediT authorship contribution statement

**Jun Luo**: Conceptualization, Methodology, Formal analysis, Data collection, Writing-original draft. **Mingzhao Xiao**: Formal analysis, Writing - review & editing. **Zhujizhaba Gama**: Data collection. **Deji Gesang**: Data collection. **Qing Liu**: Data collection. **Ying Zhu**: Formal analysis, Writing - original draft. **Lining Yang**: Formal analysis, Writing - original draft. **Dingqun Bai**: Writing - review & editing.

## Funding

This research was funded by The 10.13039/501100012166National Key Research and Development Program of China (No.2020YFC2005900) and The Key Research and Development Program Jointly Implemented by Sichuan and Chongqing (No. cstc2020jscx-cylhX002).

## Declaration of competing interest

The authors have declared no conflict of interest.

## Data availability statement

The datasets used and analyzed during the current study are available from the corresponding author on reasonable request.
