# Supplementary material for: Real-life experience of accepting assistive device services for Tibetans with dysfunction: A qualitative study
Source: Int J Nurs Sci. 2022 Dec 21;10(1):104–10. doi: 10.1016/j.ijnss.2022.12.005 (PMC9969061; doi:10.1016/j.ijnss.2022.12.005)
Supplement: Multimedia component 1 [file mmc1.docx]

藏族功能障碍者接受辅助器具服务的真实体验

罗君, 嘎玛珠吉扎巴, 格桑德吉, 刘庆, 朱莹, 杨力凝, 白定群,肖明朝

【摘要】

**目的** 了解西藏自治区藏族功能障碍者在接受辅助器具服务过程中的真实体验，为服务质量改善和政策制定提供参考。

**方法** 采用半结构式个人访谈收集资料。于2021年9—12月以目的抽样法在拉萨市选取代表3类不同经济水平地区的10名藏族功能障碍者参与研究。采用Colaizzi七步法进行资料分析。

**结果** 结果呈现了3个主题和7个子主题：认同辅助器具带来的切实益处（提升功能障碍者自理能力；协助家庭成员照护工作，促进家庭关系和谐）；面临的问题及负担（专业服务获取困难且流程繁琐；不会正确使用；心理负担：害怕跌倒和污名化）；需求及期望（提供社会支持，降低使用成本；提升基层无障碍设施可及性，改善辅助器具使用环境）。

**结论** 正视藏族功能障碍者在接受辅助器具服务过程中面临的问题及挑战，关注功能障碍群体的真实体验，提出有针对性地改善和优化用户体验的建议，可为未来干预研究及相关政策制定提供参考及依据。

【关键词】经验；少数民族；定性研究；自助装置

通信作者：肖明朝，E-mail:201606@hospital.cqmu.edu.cn
